# Health-care burden related to respiratory syncytial virus in a resource-constrained setting: a prospective observational study

**DOI:** 10.1016/S2214-109X(25)00048-8

**Published:** 2025-05-21

**Authors:** Senjuti Saha, Sudipta Saha, Naito Kanon, Yogesh Hooda, Mohammad Shahidul Islam, Shuborno Islam, Zabed Bin Ahmed, Sheikh Wasik Rahman, Md Jahangir Alam, Ataul Mustufa Anik, Probir K Sarkar, Mohammed Rizwanul Ahsan, Md Ruhul Amin, Samir K Saha

**Affiliations:** a Child Health Research Foundation, Dhaka, Bangladesh; b Department of Social and Behavioral Sciences, Harvard T H Chan School of Public Health, Boston, MA, USA; c Department of Pediatric Respiratory Medicine (Pulmonology), Bangladesh Shishu Hospital and Institute, Dhaka, Bangladesh; d Department of Emergency, Observatory and Referral, Bangladesh Shishu Hospital and Institute, Dhaka, Bangladesh; e Department of Microbiology, Bangladesh Shishu Hospital and Institute, Dhaka, Bangladesh

## Abstract

**Background:**

Respiratory syncytial virus (RSV) is a leading cause of paediatric hospital admissions worldwide, straining health systems. A lack of data on the burden of RSV infections and the impact on health systems in resource-limited settings hinders evidence-based policy decisions. Here, we aimed to assess RSV's burden on the health system in Bangladesh.

**Methods:**

From January to December, 2019, we conducted a prospective study at Bangladesh's largest paediatric hospital among children aged 0–59 months admitted with a possible respiratory infection, as guided by the WHO RSV hospital-based surveillance case definition. Outcomes for RSV-positive children younger than 5 years were analysed. We also followed up outcomes of children denied hospitalisation due to bed shortages. Adjusted hazard ratios for children denied admission versus admitted were estimated using survival analysis. Monte Carlo simulations with a queueing model were used to estimate the effects of RSV prefusion F maternal vaccine or nirsevimab on admission denials and mortality.

**Findings:**

Of 40 664 children admitted, 31 692 were younger than 5 years; 19 940 were in study wards. Among 7191 admitted with possible respiratory infections, 6149 (85·5%) had nasopharyngeal swabs taken, with 1261 (20·5%) testing RSV-positive. The median age of children who tested positive for RSV was 3·0 months (IQR 1·0–8·0), with a median hospital stay of 5 days (IQR 4–8); 24 (1·9%) of 1261 died in hospital. 8274 (5·5%) of 151 110 bed days were for children who were positive for RSV. Additionally, of 9169 children denied admission, outcomes were tracked for 3928 and compared with 2845 admitted. The hazard ratio for death was 1·56 (95% CI 1·34 to 1·81) for children denied versus admitted, being highest for neonates at 2·27 (1·87 to 2·75). RSV prefusion F maternal vaccine or nirsevimab could have reduced denials by 677 (95% prediction interval 63 to 1347) and 1289 (684 to 1865), respectively, potentially preventing 130 (–60 to 322) and 258 (32 to 469) deaths.

**Interpretation:**

RSV strains health care in Bangladesh, increasing mortality risks. Preventive interventions could lessen its impact, boosting health-care capacity and child health in resource-limited settings.

**Funding:**

The Bill & Melinda Gates Foundation.

## Introduction

Respiratory syncytial virus (RSV) is a leading cause of acute lower respiratory infection in children younger than 5 years. Annually, RSV infections are estimated to result in 33 million episodes, 3·6 million hospital admissions, and more than 100 000 deaths worldwide.^1^ Low-income and middle-income countries (LMICs) bear a disproportionate burden of the disease, where more than 95% of RSV-associated acute lower respiratory infection episodes and more than 97% of RSV-attributable deaths occur.^1^

Interventions such as maternal vaccines and long-acting monoclonal antibodies have the potential to reduce RSV-related morbidity and mortality.^2^, ^3^, ^4^, ^5^, ^6^ There is also growing evidence suggesting that these interventions might have broader benefits, such as reducing respiratory illnesses caused by other pathogens, and decreasing recurrent hospitalisations.^7^, ^8^, ^9^ Policy makers are grappling with how to prioritise and implement these measures, but often lack up-to-date and locally relevant epidemiological data from LMICs to guide these decisions.

In Bangladesh, a densely populated lower-middle-income country with a birth cohort of almost 3 million, the health-care system faces challenges due to the high burden of communicable and non-communicable diseases.^10^, ^11^ Data from the country's largest paediatric hospital indicate that approximately 20% of children needing hospitalisation—whether for communicable or non-communicable diseases—have to be turned away due to bed shortages.^12^ In these settings, a high RSV burden can exacerbate capacity issues while also potentially leading to adverse health outcomes for children who are denied admission. Thus, considering both the direct and indirect effects of RSV infections on child health and the broader health system when making informed policy decisions is imperative.

**Research in context**

**Evidence before this study**

On May 5, 2024, we conducted a literature review on respiratory syncytial virus (RSV)-related hospital admissions in low-income and middle-income countries (LMICs) using PubMed. We used MeSH terms such as “respiratory syncytial virus”, “children”, “disease burden”, “incidence”, and “LMICs” without restricting the search by publication date. Only articles in English were considered. Our search primarily identified studies from south Asia and sub-Saharan Africa. Among these, fewer than 100 articles provided primary data on hospital RSV burden, detecting RSV in 13–45% of hospitalised children with respiratory symptoms. A population-based study (Aetiology of Neonatal Infections in South Asia) estimated the incidence of RSV in south Asia to be 6·3 episodes per 1000 livebirths, with 7·32 episodes per 1000 livebirths specifically in Bangladesh. A hospital-based study (Pneumonia Etiology Research for Child Health) estimated that RSV was the cause of 32·2% of hospitalisations due to severe pneumonia in Bangladesh. The reported hospital case fatality rates associated with RSV ranged from 1–2%, with estimated annual management costs of RSV-associated acute lower respiratory infections between €3·47–€7·93 billion. Existing literature, however, does not discuss the indirect effects of RSV infections on health systems, such as the contribution of hospitalisations due to RSV infections to competition for limited hospital beds.

**Added value of this study**

This study uniquely quantifies the effect of RSV on hospital admissions and child mortality at Bangladesh's largest tertiary-level paediatric hospital, which frequently faces bed shortages, akin to other resource-limited settings. RSV accounted for 8274 of the total 151 110 observed bed days. Of the 1261 RSV-positive cases recorded, 24 (1·9%) patients died in the hospital. 36 deaths were recorded, including 12 post-discharge deaths, among 205 children who tested positive for RSV and who were followed up for 90 days, with common comorbidities being pneumonia, sepsis, congenital heart disease, and failure to thrive. During the study, 9169 (18·4%) of 49 833 children requiring hospitalisation (for all causes) were not admitted due to insufficient bed capacity. We followed up about 6000 children for 90 days post-hospital visit, including those admitted and those denied admission due to bed shortages. The hazard ratio for death was 1·6 for denied versus admitted children, highest within babies in their first month of life at 2·3. Our analysis suggests that the introduction of licensed preventive interventions such as RSV prefusion F maternal vaccine or nirsevimab could have reduced hospital denials by 677 and 1289, respectively, averting 130 and 258 deaths during the study year.

**Implications of all the available evidence**

Integrating our findings with the existing literature underscores the crucial role of maternal RSV vaccination and monoclonal antibodies in reducing health system burdens in LMICs. By potentially lowering hospital admissions for RSV, such interventions could enable more efficient resource management, allowing increased admissions and improved survival rates for children needing care for conditions other than RSV, especially neonates. These findings support RSV immunisations as a strategic public health measure. Furthermore, our data show the direct effect of hospital bed shortages on children's health. Given the high burden of RSV and the limited number of hospital beds relative to population sizes in many countries, our findings highlight the potential applicability of this research in other settings. This approach can be extended to understand the indirect effects of disease prevention measures and direct effects of increasing hospital beds in settings in which hospitals operate at or over capacity.

Two multicountry studies, Aetiology of Neonatal Infections in South Asia (ANISA),^13^ and Pneumonia Etiology Research for Child Health (PERCH),^14^ have highlighted RSV as a primary aetiological agent in sepsis and respiratory tract infections in Bangladesh and other countries in Asia and Africa. Although these studies underscore RSV's role in child morbidity in resource-constrained settings, gaps remain in understanding the broader effect of RSV-associated hospitalisations on health-care capacity in resource-constrained settings.

To address the gaps in data and estimate the potential effect of RSV vaccines, we aimed to quantify RSV-associated hospitalisations, bed usage, and outcomes in Bangladesh's largest paediatric hospital and assess the broader health implications for children denied admission due to bed shortages by simulating different scenarios involving RSV preventive interventions.

## Methods

### Study design and setting

We conducted a prospective observational surveillance study at Bangladesh Shishu Hospital and Institute in Dhaka, Bangladesh. This hospital, with its 653-bed capacity (2019), is Bangladesh's largest paediatric hospital, offering primary to tertiary care for patients aged up to 18 years. Admission decisions for the inpatient department are made by physicians in the emergency room.

This study had two main components: active surveillance within the inpatient department to detect RSV infections; and active surveillance of health outcomes of patients who, despite needing hospitalisation, were denied admission due to bed shortages in the inpatient department, and a control group of patients who were admitted to the inpatient department for any reason.

This study was approved by the ethical review board of the Bangladesh Shishu Hospital and Institute. For children admitted to the hospital, written consent was obtained from parents or guardians for nasopharyngeal sample collection, testing, and inclusion in the study. For children denied hospitalisation, data on their health outcomes were obtained from caregivers over the telephone after obtaining verbal consent.

### RSV surveillance in the inpatient department

Children aged 0–59 months admitted to the wards selected for the study were evaluated by study physicians. Inclusion criteria for this study were children hospitalised with a possible respiratory infection as guided by the WHO RSV hospital-based surveillance case definition (appendix 1 p 3).^15^ These study wards contained 414 beds; the remaining 239 beds of the total 653 were not included because they were in wards that do not admit patients with possible infectious diseases (eg, surgery, nephrology, oncology, and cardiology wards). Nasopharyngeal swabs were collected by trained nurses from eligible children except those requiring support from equipment such as high-flow nasal cannulas, continuous positive airway pressure machines, headboxes, or incubators (appendix 1 p 25), and were tested using quantitative PCR (appendix 1 p 5).

### Surveillance of children denied admission in the emergency room

Upon arrival at Bangladesh Shishu Hospital and Institute, families with children potentially needing hospitalisation are triaged in the emergency room. Beds are available on a first come, first serve basis. If the physician decides that the child requires hospitalisation, but no beds are available, families are referred or advised to seek care elsewhere. During this study, when a child could not be admitted due to bed shortages, despite requiring hospitalisation (referred to here as denied), trained research assistants documented the emergency room physician's diagnosis for the patients and collected the parents’ or a family member's contact information during their hospital visit (between 8:00 and 21:00) and obtained permission to call them later (appendix 1 p 3). Here, denials refer only to denial at Bangladesh Shishu Hospital and Institute and do not indicate that children were unable to receive care at a different hospital.

### Health outcome follow-up

Among the children younger than 5 years who were denied admission due to bed shortages and whose contact information was obtained, families were selected using a computer-based randomisation algorithm and contacted via telephone approximately 2 weeks after denial of admission, and the child's health status (ie, alive or deceased), and date of death (if applicable) were recorded upon obtaining verbal consent on study participation and data collection. If the child was alive during the 2-week follow-up, a second follow-up call was made 3 months after denial of admission. In parallel, from the cohort of children younger than 5 years who were admitted during the same time, children were randomly chosen for follow-up in the same manner. Survival curves were computed using the Kaplan–Meier method and crude and adjusted hazard ratios were calculated using Cox proportional hazards models (appendix 1 p 6).

### Estimated impact of RSV preventive interventions

We used queueing theory^16^ and Monte Carlo simulations to understand how a reduction in RSV cases requiring admission might affect: the strain on hospital capacity, the overall denial of patients seeking admission, and mortality. Our model algorithm was based on the admissions process described above and simulated daily admissions and discharges through the hospital, given observed and hypothetical caseloads. Details of the simulations are provided in appendix 1 (pp 7–18). Different scenarios with various reductions in the number of RSV cases were simulated, including for the recently approved prefusion F maternal vaccine (RSVpreF), and the long-acting monoclonal antibody, nirsevimab.

### Role of the funding source

The funder of the study had no role in study design, data collection, data analysis, data interpretation, writing of the report, or the decision to submit this manuscript for publication.

## Results

**Figure 1:**
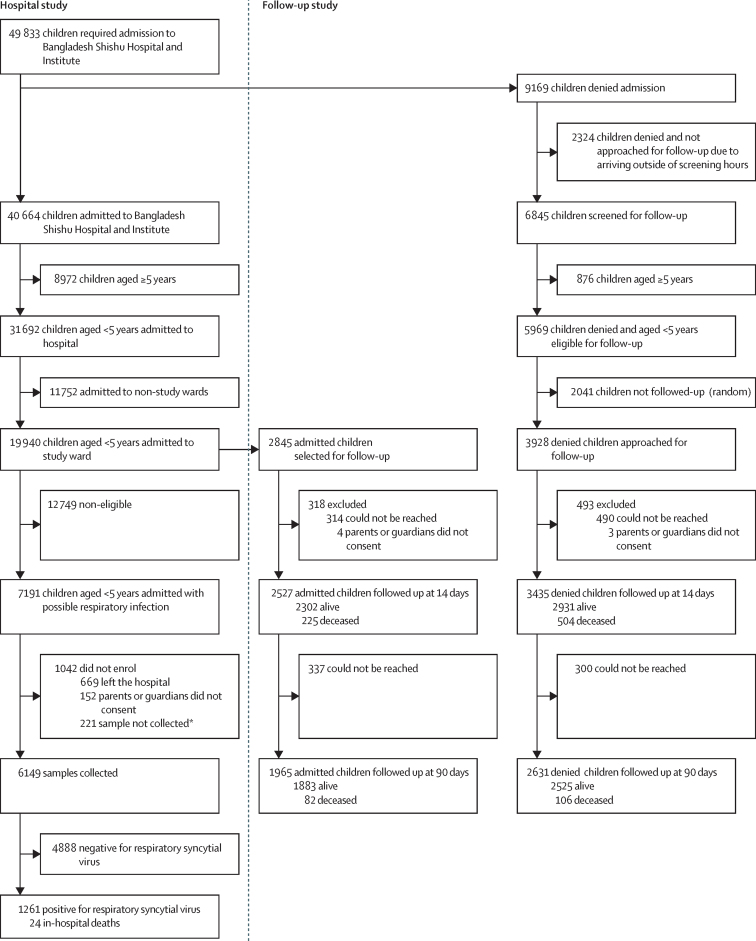
Flowchart of sample collection and patient follow-up The figure illustrates the process of sample collection and subsequent patient follow-up for children younger than 5 years admitted to Bangladesh Shishu Hospital and Institute or denied admission due to the unavailability of beds. *Reasons why samples could not be collected are given in appendix 1 (p 25).

From January to December, 2019, 49 833 children sought care at Bangladesh Shishu Hospital and Institute and were advised admission, of whom 40 664 were able to be admitted based on available beds (figure 1). 31 692 of them were younger than 5 years, and of them, 19 940 were admitted to the screening study wards (table 1). Of those admitted, 7191 met the RSV hospital-based surveillance case definition, and a sample was successfully collected from 6149 (85·5%) of these children. 1042 children were not enrolled: 152 because parents or guardians did not consent, 669 because they were discharged before nasopharyngeal swabs could be collected, and 221 because sample collection was not possible for various reasons, such as the use of a high-flow oxygen cannula or being in a special ward (eg, intensive care unit; figure 1; appendix 1 p 25). 5610 (91·7%) of the 6119 samples, for which a time of collection was available, were collected within 72 h of hospitalisation (appendix 1 p 26).

**Table 1:** Clinical attributes of children younger than 5 years admitted to study wards by RSV status

		**Overall (N=19 940)**	**RSV positive (n=1261)**	**RSV negative (n=4888)**	**Eligible but untested (n=1042)**	**Not eligible (n=12 749)**	**p value**
Sex							<0·0001
	Male	12 358 (62·0%)	875 (69·4%)	3133 (64·1%)	650 (62·4%)	7700 (60·4%)	**..**
	Female	7582 (38·0%)	386 (30·6%)	1755 (35·9%)	392 (37·6%)	5049 (39·6%)	..
Age, months		5 (0–16)	3 (1–8)	2 (0–10)	0 (0–6)	7 (0–21)	<0·0001
	<2 weeks	5448 (27·3%)	72 (5·7%)	1486 (30·4%)	466 (44·7%)	3424 (26·9%)	..
	2 weeks–3 months	3955 (19·8%)	625 (49·6%)	1214 (24·8%)	262 (25·1%)	1854 (14·5%)	..
	4–6 months	1648 (8·3%)	184 (14·6%)	500 (10·2%)	78 (7·5%)	886 (6·9%)	..
	7 months–2 years	5693 (28·6%)	312 (24·7%)	1194 (24·4%)	185 (17·8%)	4002 (31·4%)	..
	2–5 years	3196 (16·0%)	68 (5·4%)	494 (10·1%)	51 (4·9%)	2583 (20·3%)	..
In-hospital case fatality (%)		1266 (6·3%)	24 (1·9%)	372 (7·6%)	291 (27·9%)	579 (4·5%)	<0·0001
Length of stay, days		4 (2–7)	5 (4–8)	6 (3–9)	2 (1–4)	4 (2–7)	<0·0001
Diagnosis clusters							
	Respiratory manifestation	5476 (27·5%)	988 (78·4%)	2392 (48·9%)	475 (45·6%)	1621 (12·7%)	<0·0001
	Gastrointestinal manifestation	2498 (12·5%)	45 (3·6%)	170 (3·5%)	24 (2·3%)	2259 (17·7%)	<0·0001
	Perinatal asphyxia	2570 (12·9%)	24 (1·9%)	936 (19·1%)	285 (27·4%)	1325 (10·4%)	<0·0001
	Febrile illness	1548 (7·8%)	25 (2·0%)	209 (4·3%)	24 (2·3%)	1290 (10·1%)	<0·0001
	Preterm low birthweight	1136 (5·7%)	19 (1·5%)	341 (7·0%)	127 (12·2%)	649 (5·1%)	<0·0001
	Systemic infections	2782 (14·0%)	131 (10·4%)	831 (17·0%)	269 (25·8%)	1551 (12·2%)	<0·0001
	Other*	12 817 (64·3%)	466 (37·0%)	3064 (62·7%)	643 (61·7%)	8644 (67·8%)	<0·0001

Data are n (%) or median (IQR) unless otherwise specified. p values were computed with χ^2^ tests for categorical variables (sex, case fatality rate, and diagnosis) and Kruskal–Wallis tests for the continuous variables (age and length of stay). RSV=respiratory syncytial virus.

* A list of diagnoses that could be included in the other category is provided in appendix 1 (p 19).

All 6149 samples were tested for the presence of RSV using quantitative PCR, and of them 1261 (21%) samples tested positive. Assuming 1042 untested eligible cases among 7191 had the same positivity as those tested, the overall proportion of all hospital admissions that were RSV-positive was 4·65% or 465 RSV cases (95% CI 443–489) per 10 000 hospitalisations in those younger than 5 years.

**Figure 2:**
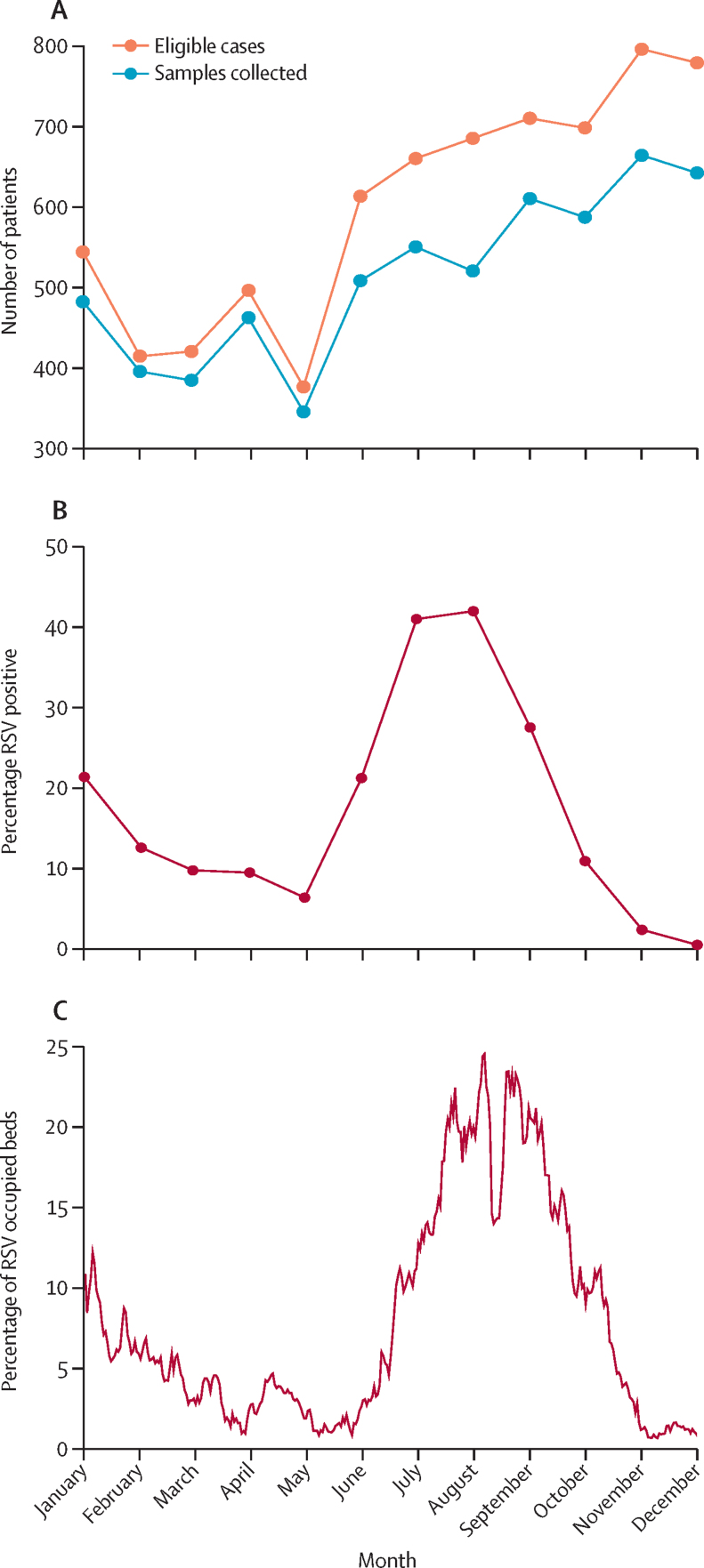
RSV infections and bed occupancy in Bangladesh Shishu Hospital and Institute in 2019 (A) The total number of patients meeting the eligibility criteria of the study each month, and the number of samples collected from these patients. (B) The percentage of occupied beds that were RSV cases. (C) The percentage of beds occupied by children with RSV across different months. The dip in bed occupancy seen in mid-August is likely because of the Eid-ul-Adha holidays. RSV=respiratory syncytial virus.

882 (69·9%) of the 1261 total RSV-positive cases were enrolled in the study during the 4 months from June to September (figure 2). On average, 17·0%, 20·2%, and 15·4% of all occupied beds each day were occupied by RSV cases during the months of July, August, and September, respectively (figure 2).

Of the positive cases, 386 (30·6%) were female. The median age of children who tested positive for RSV was 3 months (IQR 1–8) and 881 (70·0%) of the children were within their first 6 months of life (table 1; appendix 1 p 15). 214 (17·0%) of infections occurred in the first 4 weeks of life, highlighting the vulnerability of very young babies to RSV. The median hospital stay for children who tested positive for RSV was 5 days (IQR 4–8), while the median stay of all hospitalised children was 4 days (2–7). Throughout the year, RSV infections accounted for 8274 (5·5%) of the total 151 110 bed days observed.

**Figure 3:**
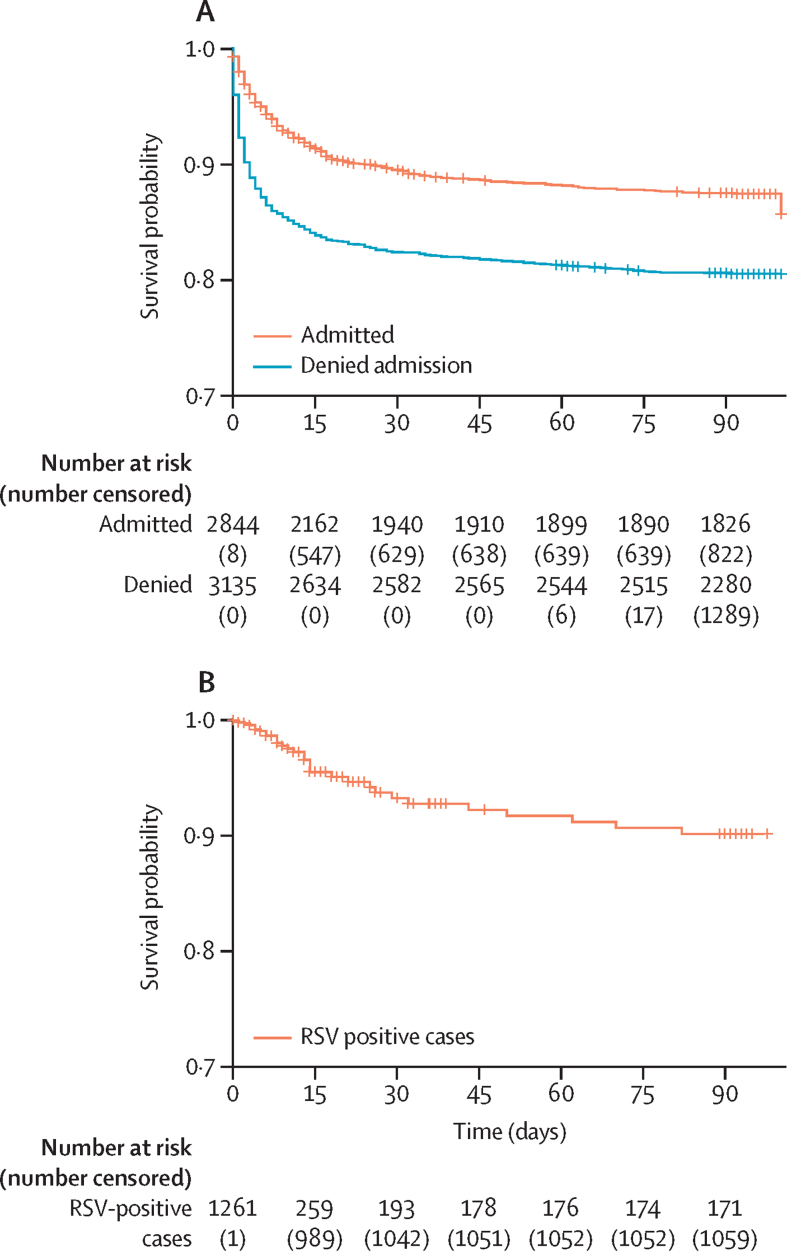
Survival analysis of patients followed up (A) Survival probabilities of patients denied admission (blue line) and those admitted (orange line). Cases were censored at discharge if there was no follow-up or at the last successful follow-up. (B) Survival curve for only RSV-positive cases: the 24 cases that resulted in in-hospital deaths, alongside the additional 205 cases that were followed up, of whom 18 subsequently died. Numbers at risk at different timepoints are provided below the graphs.

During the study period, of the 49 833 children requiring hospitalisation, the hospital was unable to admit 9169 (18·4%) patients due to bed shortages (figure 1). Of them, we approached and collected contact information of 5969 families for follow-up; 3200 patients were not approached because they either were 5 years and older (n=876) or came to the hospital at night when the study research assistants were unavailable (n=2324; figure 1). Of the families who provided contact information for telephone follow-up, a random selection of 3928 (42·8%) patients were contacted for follow-up. Of these, 3435 (87·4%) were successfully contacted, provided verbal consent over the telephone to participate, and were followed up at 2 weeks (figure 1). During the first follow-up, 504 (14·7%) were reported to have died. Of the 2931 patients alive, 2631 (89·8%) could be subsequently contacted during the 3-month follow-up, and an additional 106 (3·6%) had died. The survival probability at the end of follow-up was 0·805 (95% CI 0·791–0·819) for denied patients (figure 3).

2845 admitted children younger than 5 years were randomly selected for follow-up during the same time. Of them, 2527 (88·8%) were successfully followed up at 2 weeks over the telephone with consent. During the first follow-up, 225 (8·9%) were reported to have died (table 2). Of the 2302 patients alive, 1965 (85·3%) could be subsequently contacted during the 3-month follow-up, among whom an additional 82 (4·2%) had died (figure 1). The survival probability at the end of follow-up was 0·874 (95% CI 0·861–0·887) among those admitted (figure 3).

**Table 2:** Clinical attributes and outcome of participants admitted or denied admission who were followed up

		**Overall (N=6773)**	**Admitted (n=2845)**	**Denied (n=3928)**	**p value**
Sex					0·5077
	Male	4191 (61·9%)	1774 (62·4%)	2417 (61·5%)	..
	Female	2582 (38·1%)	1071 (37·6%)	1511 (38·5%)	..
Age, months		3 (0–12)	4 (0–15)	3 (0–11)	0·0069
	<2 weeks	2022 (29·9%)	809 (28·4%)	1213 (30·9%)	<0·0001
	2 weeks–3 months	1425 (21·0%)	595 (20·9%)	830 (21·1%)	..
	4–6 months	622 (9·2%)	226 (7·9%)	396 (10·1%)	..
	7 months–2 years	1869 (27·6%)	764 (26·9%)	1105 (28·1%)	..
	2–5 years	835 (12·3%)	451 (15·9%)	384 (9·8%)	..
Diagnosis cluster		N=6209	n=2429	n=3780	
	Respiratory manifestation	1664 (26·8%)	552 (22·7%)	1112 (29·4%)	<0·0001
	Gastrointestinal manifestation	543 (8·7%)	250 (10·3%)	293 (7·8%)	0·0006
	Perinatal asphyxia	772 (12·4%)	281 (11·6%)	491 (13·0%)	0·106
	Febrile illness	267 (4·3%)	160 (6·6%)	107 (2·8%)	<0·0001
	Systemic infections	577 (9·3%)	226 (9·3%)	351 (9·3%)	1·0
	Preterm low birthweight	418 (6·7%)	114 (4·7%)	304 (8·0%)	<0·0001
	Other*	1968 (31·7%)	846 (34·8%)	1122 (29·7%)	<0·0001
14-day mortality		729 (12·2%)	225 (8·9%)	504 (14·7%)	<0·0001
90-day mortality		188 (4·1%)	82 (4·2%)	106 (4·0%)	0·8659

Data are n (%) or median (IQR) unless otherwise specified. p values were computed with χ^2^ tests for categorical variables (sex, diagnosis, and mortality) and the Kruskal–Wallis test for age. Diagnoses could not be collected for 564 (8·3%) of participants, 14-day mortality was missing for 811 (12·0%) due to being lost to follow up, and 90-day mortality was missing for 2177 (32·1%) due to being lost to follow-up or mortality at 14 days. 90-day mortality is among those alive at 14 days and successfully followed-up.

* A list of diagnoses that could be included in the other category is provided in appendix 1 (p 19).

Overall comparison between the two groups revealed a hazard ratio (HR) of 1·69 (95% CI 1·48–1·94) for death in children who were denied admission compared with children who were admitted (appendix 1 p 27). The HR was 1·56 (95% CI 1·34–1·81) in models adjusted for age, diagnosis (categorised as presented in table 2), and sex. Models stratified by age groups showed that the HR for death was significantly greater among babies who were denied admission in their first month of life (HR 2·27 [95% CI 1·87–2·75]) but not in other age groups. When stratified by diagnosis, HR of death was significantly greater among those with perinatal asphyxia (2·05 [1·59–2·65]), and preterm low birthweight (2·78 [1·99–3·87]).

Of the 1261 children with RSV recorded in this study, 24 (1·9%) died during their stay at the hospital (table 1). The median age of these children was 130 days (IQR 77–175). Although the study was not designed to specifically follow up children with RSV, of the 2527 admitted children randomly selected and successfully followed up, there were 205 RSV-positive cases. Of these, 18 children died within the 90-day follow-up period, six of whom were also recorded as one of the 24 hospital deaths. This brought the total number of deaths recorded in this study to 36. Details of all cases are provided in the appendix 1 (pp 28–30). The overall median age was 116·5 days (IQR 69·5–220·3). Considering both in-hospital case fatality of all admitted children who tested positive for RSV and mortality during follow-up of a subset of cases, survival decreased substantially over time, with a 90-day survival probability of 0·901 (95% CI 0·865–0·938) at the end of the follow-up period (figure 3). The most common final diagnosis was pneumonia (22 cases), frequently associated with additional conditions such as sepsis (nine cases) and congenital heart disease (12 cases). RSV cannot be attributed as the underlying cause of death for all these cases, as this study was not designed to establish a causal link between RSV and mortality.

**Figure 4:**
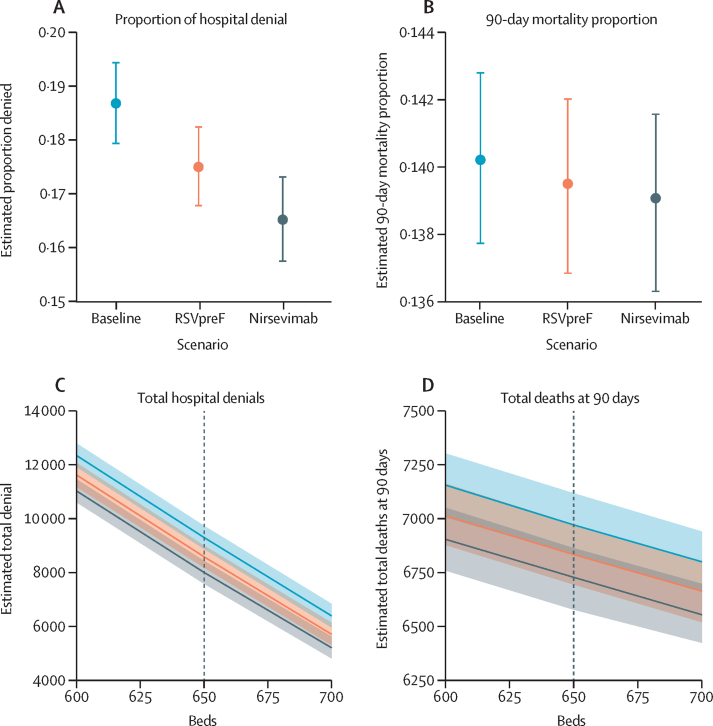
Effect of RSV preventive interventions on hospital use and 90-day mortality rates of all children who require admission at Bangladesh Shishu Hospital and Institute Effects of RSVpreF and nirsevimab on RSV admissions, showing its impact on the proportion of hospital denial (A), 90-day mortality proportion (B), total hospital denials (C), and total deaths at 90 days (D) among children younger than 5 years. The plots C and D compare mortality against the effective increase in available beds, showing how reducing RSV cases is akin to increasing bed capacity. Solid lines in (A) and (B) and shaded areas in (C) and (D) represent prediction intervals. The dotted line in (C) and (D) represent the current bed number at Bangladesh Shishu Hospital and Institute. RSV=respiratory syncytial virus. RSVpreF=RSV prefusion F maternal vaccine.

In Monte Carlo simulations of hospital admissions and denials for the baseline scenario, based on the empirical data and a 650-bed capacity, a median of 49 726 children required admissions (95% prediction interval [PI] 49 355 to 50 082), and 9283 (8861 to 9714) cases were denied admission over the year. This was in concordance with the true burden of total cases requiring admission and denials—49 833 and 9169, respectively. In the scenario in which RSVpreF is introduced with an estimated 38·3% reduction in RSV cases requiring hospitalisation (appendix 1 pp 13–14), 48 972 (95% PI 48 632 to 49 317) patients required admission, and 8571 patients (8173 to 8980) were denied admission—a 1·19 percentage point reduction in the median proportion of cases that were denied admissions across simulations (figure 4A). In the nirsevimab scenario, with an estimated 69% reduction in RSV cases requiring hospitalisation (appendix 1 pp 13–14), 48 367 (95% PI 48 006 to 48 731) patients required admission, and 7991 patients (7574 to 8416) were denied admission—a 2·2 percentage point reduction (figure 4A). This corresponded to RSVpreF or nirsevimab reducing denials by 677 (95% PI 63 to 1347) and 1289 (684 to 1865), respectively. Figure 4B shows the estimated effects of these interventions on overall mortality. Figures 4C and 4D compare these interventions with different bed capacities, showing how reducing RSV cases and increasing capacity produce similar effects. Overall, there were 6970 deaths (95% PI 6832 to 7117) in the baseline scenario, 6832 deaths (6693 to 6973) in the RSVpreF scenario, and 6727 deaths (6576 to 6862) in the nirsevimab scenario. This corresponded to an estimated median difference in deaths of 130 (–60 to 322) and 258 (32 to 469), respectively. Supplementary analyses suggested a linear relationship between the magnitude of RSV burden reduction and denial rates, holding other parameters of the system constant (appendix 1 p 17).

## Discussion

Informed decision making on interventions to prevent RSV infections in young infants requires contemporary, locally relevant epidemiological data. Our study shows RSV's substantial impact on health-care resources in Bangladesh, a country with a high infectious disease burden and demand for paediatric care amid resource limitations.^12^

Our study recorded a 21% RSV positivity rate among 6149 patients admitted with respiratory illnesses to Bangladesh's largest paediatric hospital, corresponding to 465 RSV cases per 10 000 admissions of children younger than 5 years. The median age of children with confirmed RSV was 3 months. These findings align with those of the PERCH study, which reported that 31·2% of children hospitalised with respiratory infections in Bangladesh tested positive for RSV.^14^, ^17^

The in-hospital case fatality for RSV-positive cases was 1·9%. Among 205 randomly selected children with RSV followed up for up to 90 days post-hospitalisation, 18 deaths were recorded. However, these children had notable comorbidities, and this study was not designed to attribute these deaths solely to RSV infection. The median age of all recorded RSV-associated deaths was 116 days (or 3·8 months). In the Child Health and Mortality Prevention Surveillance (CHAMPS) study, a community-based study not limited to in-hospital deaths, RSV was found in 5·5% of all deaths, with variability across countries: 9·7% in Mali, 10·7% in Ethiopia, and 2% in Bangladesh.^18^ Half of the RSV-associated deaths in the CHAMPS study occurred in infants younger than 6 months, highlighting the vulnerability of young infants to severe RSV outcomes.

Beyond the direct effect of RSV infections, our findings also show the considerable burden of infections on the health-care system. For at least 3 months during the study year, children with RSV occupied more than 20% of the beds under observation. The median hospital stay for children with RSV was 5 days—longer than the overall median length of stay of 4 days for all admitted patients. In 2019, RSV cases accounted for 8274 of the total 151 110 bed days observed. Despite being the largest paediatric tertiary hospital with 653 beds, Bangladesh Shishu Hospital and Institute faces bed shortages, often forcing clinicians to deny admission to sick children.^12^ We followed more than 5000 admitted and denied children for up to 90 days to determine the difference in health outcomes and the wider effects of high-burden infections such as RSV on the health-care system. The cumulative mortality proportion for children denied admission was 19·5%, substantially higher than the 12·6% among those admitted. The survival probability at the end of follow-up was 0·874 among those admitted, and 0·805 among those denied admission.

The HR for death was 1·56 among children denied admission compared with those admitted, adjusted for age, diagnosis, and sex. The highest mortality risk was observed in neonates requiring hospitalisation within their first month of life, with a HR of 2·27. This disparity highlights the essential role of timely health-care access in determining child survival outcomes. Monte Carlo simulations of a queueing model helped us characterise hypothetical scenarios with different bed capacities and numbers of patients requiring admission. Using a RSVpreF is estimated to have led to 130 fewer deaths and using a nirsevimab monoclonal antibody is estimated to have led to 258 fewer deaths during the study year among patients requiring admission to the hospital. Although the variability of these estimates was large and overlapping, the differences in admission denial rates between scenarios were substantial.

Bed shortages are a pressing issue across many LMICs. According to World Bank Open Data, in Bangladesh, the bed rate of 0·8 per 1000 population is notably lower than in high-income countries, such as 2·9 in the USA and 2·5 in the UK. With limited infrastructure, preventive strategies are essential. Although vaccine introductions and health system improvements since 2000 have reduced mortality in children younger than 5 years in Bangladesh (as shown by the Sustainable Development Goals tracker), the rate remains high at 33 deaths per 1000 livebirths, highlighting the need for more robust disease prevention. Given the substantial proportion of beds occupied by children with RSV during peak periods, an RSV maternal vaccine could substantially improve bed availability for critically ill infants. Additionally, administration of monoclonal antibodies, such as nirsevimab,^3^ in neonates and infants can also be effective, particularly given high preterm birth rates in Bangladesh.^19^ However, cost and logistical challenges complicate the adoption of monoclonal antibody treatments in LMICs such as Bangladesh.^20^, ^21^

Global data from various countries have shown shifts in RSV outbreak patterns during and after the COVID-19 pandemic.^22^ While this study was conducted in 2019, before the COVID-19 pandemic, reports from 2022 and 2023 from Bangladesh suggest that RSV cases have returned to pre-pandemic levels. A report from the Government of Bangladesh highlights a peak in RSV cases among children younger than 5 years from October, 2022, to March, 2023, with an average positivity rate of 49% in severe acute respiratory infection cases during that period.^23^ Another study examining RSV-associated deaths from August, 2009, to March, 2022, found no significant difference in RSV detection rates among deaths before and during the COVID-19 pandemic.^24^

This study has certain limitations. It was conducted in a single hospital, potentially limiting generalisability. MultiFsite research could offer a more comprehensive view of the RSV burden across Bangladesh and south Asia. However, as the largest provider of tertiary paediatric care in the country, this hospital's data are still highly informative. In addition, data collection was limited to 1 year, meaning that the simulation could only account for RSV's 2019 seasonal outbreak pattern. Unlike countries in temperate regions, with well defined RSV seasons, equatorial countries such as Bangladesh experience varied RSV peaks—sometimes in winter, summer, or even twice a year—making multi-year data crucial for modelling seasonal variability.^25^, ^26^ Additionally, RSV peaks can overlap with other endemic diseases, such as dengue^27^ and typhoid,^28^ which have unpredictable seasonal patterns, complicating generalisable pattern identification even with extended data. The study also did not assess concurrent infections with RSV or confirm deaths directly attributed to RSV. Furthermore, sample attrition and assumptions in the simulation models could introduce bias. Although we achieved an approximately 67% follow-up rate for both admitted and denied cases, those lost to follow-up could differ in important ways. During sampling, nasopharyngeal swabs were not collected from children on extensive support, such as high-flow oxygen, possibly underestimating the most severe RSV cases with adverse outcomes. In our simulations, we assumed non-study wards had similar seasonal admission patterns and that overnight denials, not included, were similar to daytime ones. However, children presenting at night, when research assistants were unavailable, might represent a sicker cohort, as emergencies often occur during these hours. This could result in an underestimation of mortality risks associated with hospital denials. As we did not assess care seeking after denial from Bangladesh Shishu Hospital and Institute, we cannot disentangle the effect of treatment delay or of an inability to access hospital care. Finally, human factors, such as rationing admission decisions based on clinicians’ understanding of disease severity, could have influenced their admission decisions during times of bed scarcity even though such decisions should theoretically be unaffected by such constraints.

Despite the limitations, the global health implications of this study are substantial. This study underscores the crucial role of RSV preventive interventions, such as vaccines and monoclonal antibodies, in reducing the disease burden in resource-limited settings. By preventing RSV-related hospitalisations, vaccination programmes could alleviate seasonal surges in bed occupancy, providing much-needed relief to overstretched health-care systems. Additionally, our findings highlight the broader issue of hospital bed shortages, which remains a major barrier to effective care in many LMICs. Our results support the prioritisation of RSV prevention programmes and expansion of hospital infrastructure to address persistent capacity challenges, improve health-care access, and enhance child health outcomes in low-resource settings.

### Contributors

### Equitable partnership declaration

### Data sharing

The study's de-identified dataset is available from the corresponding author upon reasonable request.

## Declaration of interests

We declare no competing interests.

## Supplementary Materials

Supplementary appendix 1

Equitable Partnership Declaration
