# Supplementary material for: Health-care burden related to respiratory syncytial virus in a resource-constrained setting: a prospective observational study
Source: Lancet Glob Health. 2025 May 21;13(6):e1072–81. doi: 10.1016/S2214-109X(25)00048-8 (PMC12100462; doi:10.1016/S2214-109X(25)00048-8)
Supplement: Supplementary appendix 1 [file mmc1.pdf]

THE LANCET
Global Health

Supplementary appendix 1

This appendix formed part of the original submission and has been peer reviewed. We post it as supplied by the authors.

Supplement to: Saha Se, Saha Su, Kanon N, et al. Health-care burden related to respiratory syncytial virus in a resource-constrained setting: a prospective observational study. *Lancet Glob Health* 2025; **13:** e1072–81.

Supplementary appendix

RSV-Related Healthcare Burden: A Prospective Observational Study in a Resource-Constrained Setting

Saha et al

Table of Contents

Supplementary text descriptions of methods 3

Text S1. Enrollment of cases and follow-up 3

Text S2. Nasopharyngeal swab collection and qPCR methods 5

Text S3. Survival curve analysis 6

Text S4. Queuing theory and Monte Carlo simulation 7

Supplementary Figures 15

Figure S1. Age distribution of RSV-positive cases and length of hospital stay 15

Figure S2. Baseline results of daily number of hospital admissions and denials using Monte Carlo simulations 16

Figure S3. Daily rates of patients requiring hospital admission 17

Figure S4. Various hypothetical scenarios of RSV case reduction 18

Supplementary tables 19

Table S1. Assigned clusters of related diagnoses 19

Table S2. Reasons for sample collection failure 25

Table S3. Time of sample collection 26

Table S4. Hazard ratio (HR) of denied vs admitted participants adjusted by age, sex, and diagnosis 27

Table S5. Details of all recorded deceased RSV-positive cases 28

Supplementary References 31

Supplementary text descriptions of methods

Text S1. Enrollment of cases and follow-up

Patients admitted to the pediatric wards selected for this study were identified using the ward admission logbook. Study physicians conducted a detailed clinical examination and took a thorough medical history. Children aged 0–59 months admitted to the selected wards were evaluated using the RSV hospital-based surveillance case definition.[1] Children hospitalized with a respiratory infection, defined as having cough or shortness of breath, with an onset within the last 10 days, were eligible to be enrolled in the study. In addition, infants younger than six months were also eligible if they presented with apnoea, which is characterized by a temporary cessation of breathing from any cause and/or sepsis (defined as fever (temperature of 37·5 °C or above) or hypothermia (temperature less than 35·5 °C), with indications of shock (lethargy, fast breathing, cold skin, prolonged capillary refill, or a fast, weak pulse). Once a child met the criteria, caregivers were counseled about the study, including its objectives, procedures, and potential risks and benefits. Written consent was mandatory for participation, covering both data collection and nasopharyngeal swab sampling. Following consent, the patient was enrolled, and data was recorded using a standardized, tablet-based system. Clinical follow-up was conducted daily for each enrolled patient until discharge, referral, death, or the family left against medical advice. Physicians and research assistants documented clinical signs, laboratory findings, treatment details, and final outcomes.

For patients denied admission due to bed shortages, research assistants approached the parents upon denial in the ER, and on permission to call them later to follow up on the health status of the child, collected the child's age, family contact information. Emergency room physician's diagnosis was also collected. We were very careful about ensuring that caregivers fully understood that the research team would not offer medical assistance. If verbal permission was not provided, no data were collected. This was done as quickly as possible to ensure no delay is caused in the care-seeking process. These families were informed that their participation was

optional and would not affect their access to medical care. Selected cases were called via telephone, and verbal consent was taken during these calls.

14 days after admission or refusal, research assistants contacted the caregivers of randomly selected patients by telephone to document the child's health status. Before collecting any health information, the caregiver was reintroduced to the study, including an explanation of the study procedures. Verbal consent for the child's participation in the follow-up study was obtained before collecting any information and documented. Data were only collected for the study if the caregiver provided verbal consent for their participation in the study. Collected details included child’s health status, including symptoms, recovery progress, complications, or hospital readmissions. For children alive on Day 14, an additional follow-up was conducted on Day 90 to assess longer-term health outcomes.

Text S2. Nasopharyngeal swab collection and qPCR methods

Nasopharyngeal swab samples were collected by trained nurses using mini flocked swabs (FLOQSwab, Copan Diagnostics, California, USA) and were immediately stored in 1 ml skim milk tryptone-glucose-glycerol (STGG) media. RNA was extracted from 140 µl of the sample volume using the Qiagen QIAamp® Viral RNA Mini kit on QIAcube Connect system (Qiagen, Hilden, Germany). Subsequently, 7 µl of extracted RNA underwent qPCR with qScript XLT 1-Step RT-qPCR ToughMix (QuantaBio, Beverly, MA, USA) and previously reported RSV-specific primers and probe. Final concentrations of primers and probe were 900 nM and 200 nM, respectively, in a 20 µl qPCR reaction volume. qPCR was conducted on Applied Biosystem qPCR 7500 Fast Dx system (Thermo Fisher Scientific, Waltham, MA, USA), with cycling conditions were 50°C for 10 minutes, 95°C for 1 minute, and 40 cycles of 95°C for 10 seconds and 60°C for 1 minute. RSV-positive cases were identified based on true sigmoidal amplification curves and a cycle threshold (Ct) ≤35.

Forward primer: - 5'- GGCAAATATGGAAACATACGTGAA-3'

reverse primer: 5'-TCTTTTTCTAGGACATTGTAYTGAACAG-3'

probe: 5'-CTGTGTATGTGGAGCCTTCGTGAAGCT-3' (labeled at the 5' end with FAM and quenched at the 3'end with Black Hole Quencher-1).

Text S3. Survival curve analysis

Survival curves were computed using the Kaplan-Meier method and crude and adjusted hazard ratios were calculated using Cox Proportional-Hazards models. Date of death was collected during follow-up, or from hospital records for admitted cases. Conservative censoring assumptions were made – children denied admission and not followed-up at 14-days were considered censored at day 0, and those not followed up at 90-days were considered censored at 14-days (last successful contact). Admitted children's censoring time was dealt with similarly, except that hospital discharge (either alive or through mortality) was treated as the last available information unless there was further follow-up data. Multivariable Cox Proportional-Hazards models were used to estimate hazard ratios adjusted for diagnosis cluster (table S1, appendix page 19), age in months, and sex. We also obtained stratified estimates of hazard ratios.

Text S4. Queuing theory and Monte Carlo simulation

We used queueing theory and Monte Carlo simulations to understand how a reduction in RSV cases requiring admission may impact strain on hospital capacity, overall refusals of patients seeking admission, and mortality. Queueing theory is used across a wide range of disciplines to understand the dynamics of systems where there is a demand for services, a distribution of service times, and some limitation to service provision that results in wait times or rejection. Queueing models have been in health systems research to optimize resource allocation and system design to minimize patient wait times. [2–4]

In our simulation, the system is initialized with a fixed capacity (number of beds). The average number of patients requiring admission per day is estimated based on the observed pattern of actual daily admissions and denials. The model algorithm progresses day-by-day such that each day:

1) the number of available beds is updated based on discharges;

2) number of patients requiring admission is drawn from a Poisson distribution parameterized using the observed data;

3) all patients requiring admission are admitted unless their number exceed available beds, in which case a random subset is admitted;

4) admitted patients are assigned length-of-stays (and thus discharge dates) drawn from a negative binomial distribution parameterized using the observed data, and

5) the number of available and occupied beds are updated.

Denied and admitted children are assigned separate probabilities of death at 90 days, estimated using the fully adjusted Cox Proportional-Hazards model described above, and standardized to the distribution of covariates in the population. The number of daily denials and admissions were tracked.

Details of parameterization of the algorithm, and a visual depiction is provided below. Different scenarios with various reductions in the number of RSV cases were simulated, including for recently approved maternal vaccine, RSVpreF, and long-acting monoclonal antibodies, Nirsevimab.[4,5] For the maternal vaccine, we conservatively assumed that no preterm babies

(gestational age <37 weeks) will have maternal antibodies, and used a preterm rate of 16·2%. The efficacy used for the licensed RSV maternal vaccine in our population is calculated to be 38·2% and that for the monoclonal antibody to be 69% (full details in the next section below). The median value across 1000 simulation iterations and the 97·5th and 2.5th percentile values across the simulations are reported as the 95% prediction intervals. For percentage denials and percentage mortality (Fig 5A,B), percentages are calculated in each simulation iteration, and the median, 97·5th, and 2·5th percentile values reported as well. For comparing scenarios, simulation iterations were compared across scenarios, and the median, 97·5th and 2·5th percentiles reported.

Details of parameterization of the algorithm, and a visual depiction is provided below.

Simulation structure and algorithm:

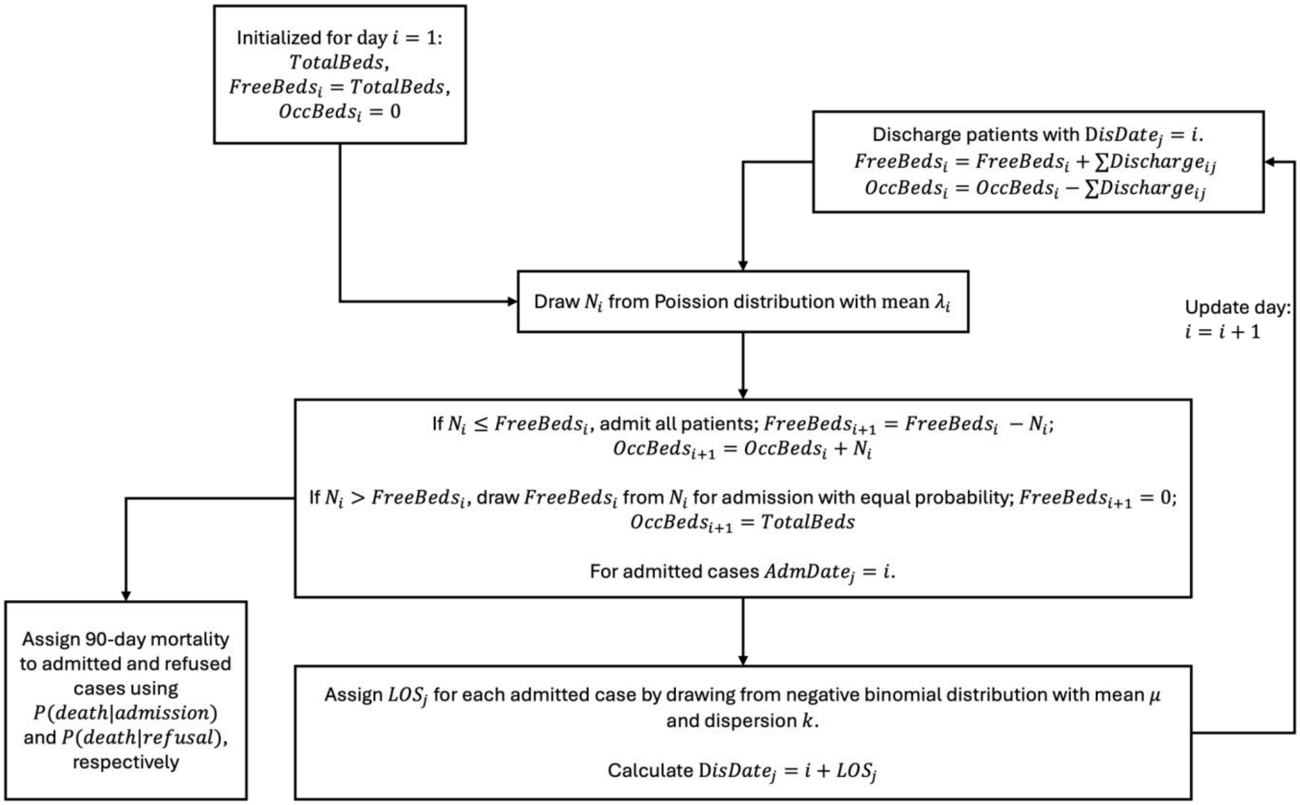

List of variables and parameters used in the simulation studies:

Variable / Parameter	Definition	Estimation / Values
$TotalBeds$	The total number of beds available for admissions to the hospital, held fixed throughout the year	Different values set for different scenarios: $TotalBeds = \{450, 500, 550, 600, 650, 700, 800, 850\}$
$FreeBeds_i$	The total number of unoccupied beds available for patients to be admitted to on a particular day i	Simulated daily based on history of admissions, and discharges based on length-of-stay of patients.
$OccBeds_i$	The total number of occupied beds available on a particular day i	Simulated daily based on history of admissions, and discharges based on length-of-stay of patients.
N_i	The total number of patients who arrive at the hospital on a particular day i requiring admission	Drawn from a Poisson distribution where $N_i \sim Poisson(\lambda_i)$
λ_i	Rate of patients arriving at hospital requiring admission on a particular day i	Estimated from the daily observed data on admissions and denials. Total admissions and total denials for 2019 were known.

		Daily admissions were known for screening wards. Daily day-time denials for <5 children were known. To match the known yearly totals and preserve the observed temporal patterns, these daily rates were scaled to match the yearly totals.<br><br>Estimated total daily denials and admissions were summed and smoothed using a 7-day moving average. These were used as λ_i (see figure S3).<br><br>Daily RSV cases were estimated by assuming that:<br>1) the positivity in the eligible and untested children was the same as in the tested children,<br>2) that the non-eligible and non-screened children did not have RSV, and<br>3) that the proportion of all cases that are RSV positive was the same among the admitted and denial cases (since no RSV testing was done among the refused). For different RSV reduction scenarios, λ_i was scaled down based on relative reduction of expected RSV cases.

$AdmDate_j$	Admission date for patient j	Simulated such that $AdmDate_j = i$ if bed is available when a patient requires admission. Further details below
LOS_j	Length-of-stay of patient j	Drawn from a negative binomial distribution with mean μ, and dispersion k
μ, k	Mean and dispersion of negative binomial distribution for length-of-stays, respectively	Estimated by fitting a negative binomial distribution to the observed length-of-stays among admitted cases. Estimates were $k = 2 \cdot 048, \mu = 5 \cdot 77$
$DisDate_j$	Discharge date for patient j	Simulated such that $DisDate_j = AdmDate_j + LOS_j$ for admitted patients. Further details below.
$Discharge_{ij}$	An indicator variable for admitted patients: 1 if patient j was discharged on day i, 0 if not	Simulated based on Dis$Date_j$. Further details below
$Death_j$	An indicator variable for 90-day mortality: 1 if patient j was alive at 90 days, 0 if not	Bernoulli draw based on admission status and parameters $P(admission), P(death\|\text{denial})$

$P(death\|admission)$ $P(death\|\text{denial})$	90-day probability of death for patients who required admission and were admitted or denied , respectively	90-day survival probability estimated for each group from survival analysis described in Methods. Estimates were: $P(death\|admission) = 0 \cdot 129$, $P(death\|\text{denial}) = 0 \cdot 189$

Vaccine Efficacy Calculations

To estimate the impact of the Pfizer maternal RSV vaccine and monoclonal antibody treatment, we calculated reductions in RSV cases based on age-specific vaccine efficacy data and RSV positivity rates.

1. **RSVpreF vaccine**:

The efficacy of the bivalent vaccine containing pre-fusion F (RSVpreF) varies by age group, according to the findings reported by Kampmann et al.[5] We specifically selected the efficacy data reported for RSV-associated hospitalization.

- <3 months: 67·7%
- 3-6 months: 56·8%
- >6 months: 0%

Age distribution of RSV positive cases in our study:

- <3 months: 55·2%
- 3-6 months: 14·6%
- >6 months: 30·2%

Reduction in RSV cases was calculated using:

$(0{\cdot}677\times0{\cdot}552) + (0{\cdot}568\times0{\cdot}146) + (0\times0{\cdot}302) = 0{\cdot}4566$ or 45·7% reduction.

After accounting for a preterm birth rate of 16·2% in Bangladesh[6] (i.e., mothers unable to receive the vaccine early enough), the overall reduction becomes: $0{\cdot}4566 \times 0{\cdot}838 = 0{\cdot}3826$ or 38·3%.

2. **Nirsevimab**:

The monoclonal antibody Nisevimab has an efficacy of 82·5% for children <1 year of age as reported by Hammitt et al.[7]

Age distribution of RSV positive cases in our study:

- <1 year: 83·7%
- >1 year: 16·3%

Reduction in RSV cases was calculated using:

0·837 × 0·825 = 0·6901 or ~69.0% reduction.

These reductions were applied to scale down the daily admission rates in the simulation to estimate the impact of RSV case reduction on hospital capacity and patient outcomes.

Supplementary Figures

Figure S1. Age distribution of RSV-positive cases and length of hospital stay

A. Age distribution of RSV cases (n = 1261) in weeks, where the green line represents RSV test positivity in different age groups, the solid orange line indicates the median age, and the dotted orange line shows the mean age. B. Length of hospital stay in days of RSV cases (n = 1261), with the solid orange line representing the median length of stay and the dotted orange line representing the mean.

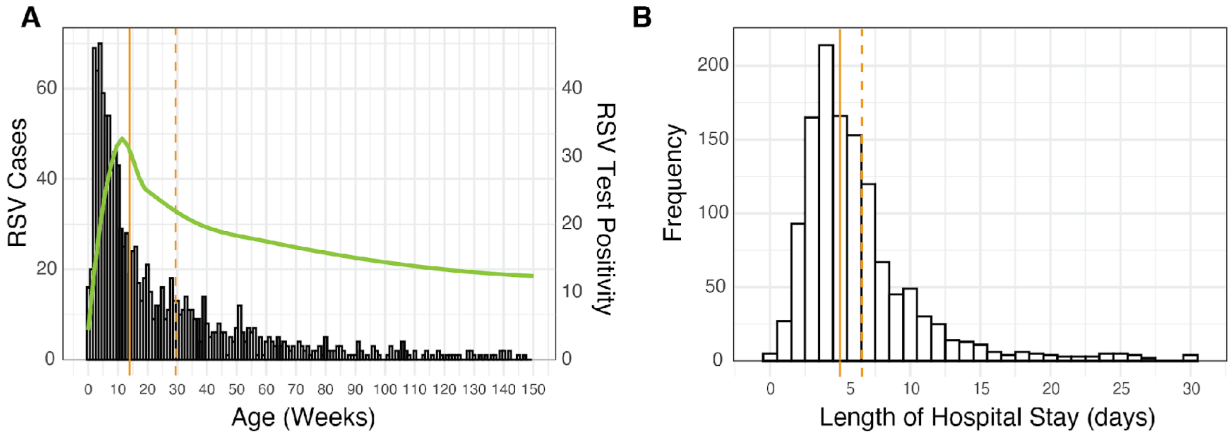

Figure S2. Baseline results of daily number of hospital admissions and denials using Monte Carlo simulations

The circles show actual data from the surveillance; the solid thick lines show the daily median across 100 simulations, and translucent lines show the 100 iterations of the simulation. Note that results in main manuscript are from 1000 iterations.

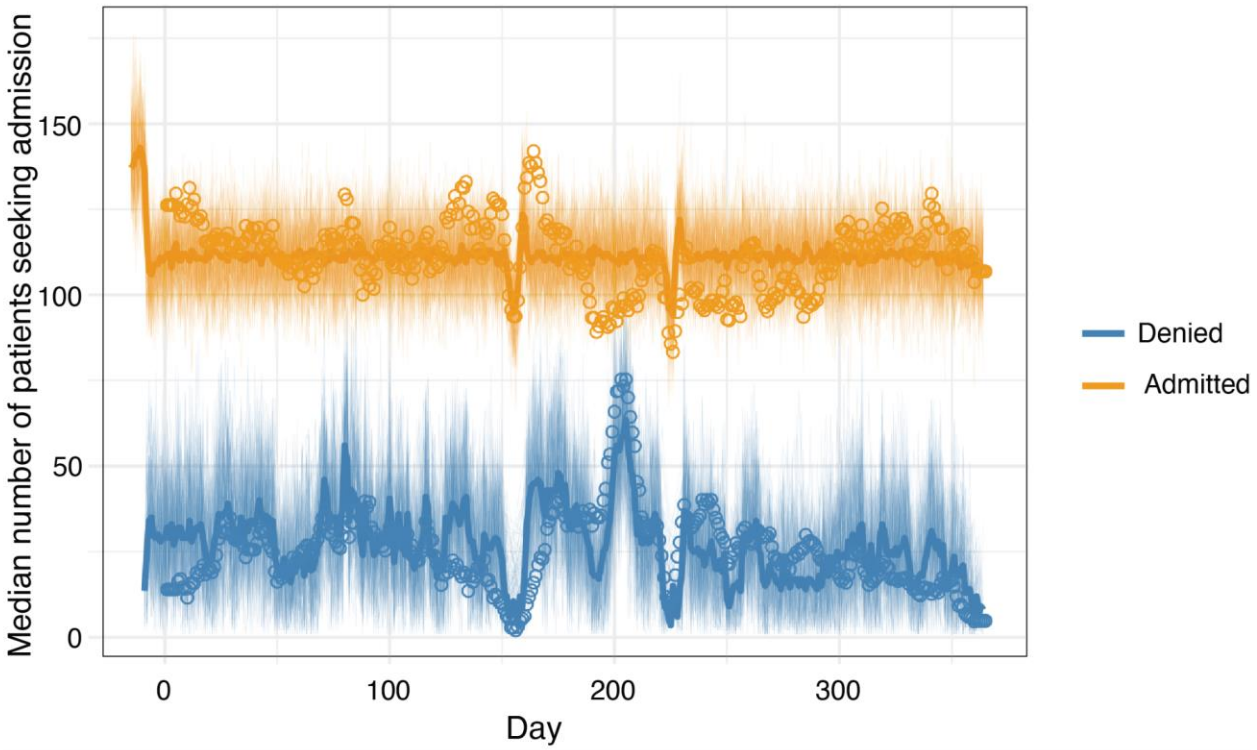

Figure S3. Daily rates of patients requiring hospital admission

Seven-day moving averages of the number of patients requiring admission per day in total, for RSV only, and without RSV. The total numbers are the daily means used for Poisson draws in the baseline scenario (λ_i). Hypothetical RSV reduction scenarios use daily means where a proportion of the RSV mean is subtracted from the daily mean.

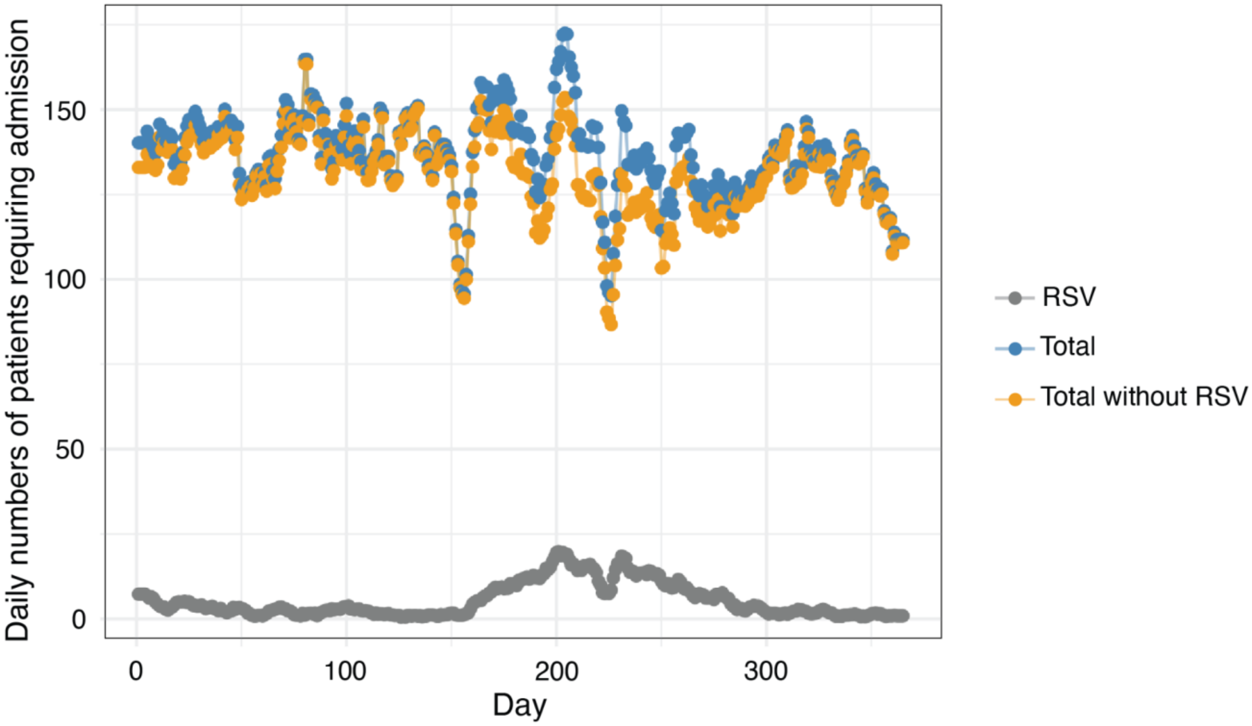

Figure S4. Various hypothetical scenarios of RSV case reduction

Estimated proportion of admissions that were denied under baseline conditions (0% RSV reduction), and for every 10% additional decrease in RSV burden. Prediction intervals and median for this figure were generated from 100 iterations.

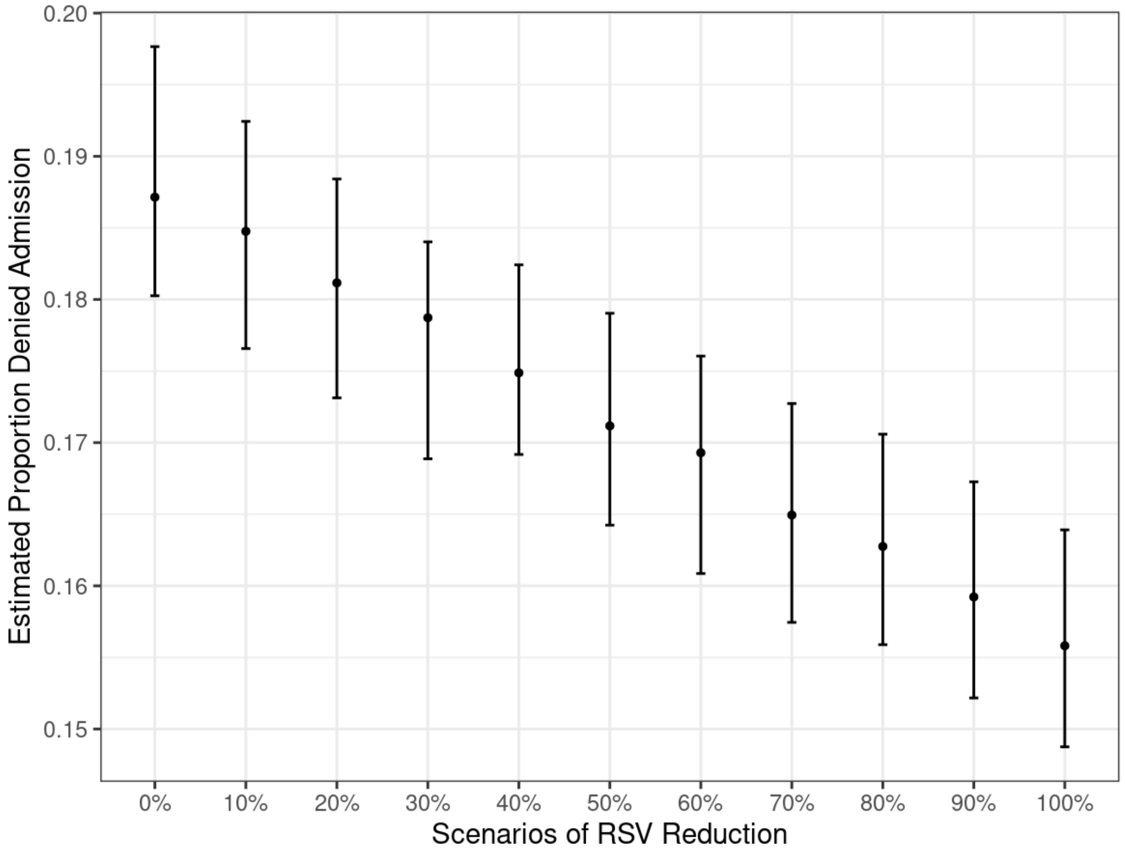

Supplementary tables

Table S1. Assigned clusters of related diagnoses

Respiratory manifestation
Pneumonia/ bronchopneumonia
Severe pneumonia
Bronchiolitis/ acute bronchiolitis
ARI/Acute respiratory infection
RDS/ Respiratory distress syndrome
Systemic infections
Septicaemia / sepsis
Neonatal sepsis
Febrile illness
Enteric fever/ typhoid fever / paratyphoid fever
Febrile convulsion / atypical febrile convulsion
Viral fever/dengue Fever
Neurological manifestation
Meningitis
Encephalitis/meningoencephalitis/encephalopathy
Seizure disorder/neonatal seizure
Epilepsy
Gastrointestinal manifestation
Acute gastroenteritis/ AGE/acute watery diarrhea
Persistent diarrhoea
Dysentery/ shigellosis/ invasive diarrhoea
Genitourinary/renal manifestation

Nephrotic syndrome
Renal failure/ kidney failure
AGN/ acute glomerulonephritis/ APSGN
Cardiovascular manifestation
Ventricular septal defect/ VSD
Tetralogy of fallot/ TOF
Other congenital heart disease
Perinatal asphyxia
Preterm low-birth weight
Neonatal jaundice
Other*

***Other may include the following diagnoses:**

Neonatal jaundice
Neurological manifestation
Genitourinary/renal manifestation
Congenital cardiovascular manifestation
Acute abdomen
ABO incompatibility/Rh incompatibility
Achondroplasia
Acute appendicitis/burst appendix
Acute Epididymoorchitis/orchitis
Acute flaccid paralysis
Acute gastritis
Acute laryngotracheobronchitis
Acute leukemia (ALL/AML)
Acute otitis media/otitis media (ASOM)
Club foot/TEV
CMV infection
Collodion baby
Congenital anomaly/ cloacal anomaly
Congenital pneumonia
Congenital rubella syndrome
Congenital syphilis
Constipation
Cough & cold/common cold
Croup
Cystic fibrosis
Cystic hygroma
Dengue fever
Dermatitis

Acute scrotum

Acute severe asthma/acute exacerbation

Acute stroke syndrome

Adrenal hyperplasia

AEFI/adverse effect following immunization

Anaemia

Anal fissure

Anorectal malformation

Anuria/oliguria

Aplastic anemia

Arthritis/rheumatoid arthritis/ JIA

Ascariasis/helminthiasis

Aspiration pneumonia

Aspiration syndrome

Ataxia/cerebellar ataxia

Bell's palsy/facial nerve palsy

Biliary atresia

Birth injury

Bladder Obstruction/urinary obstruction

Bleeding disorder

Breath holding attack

bullous disorder/epidermolysis Bullosa

Burn/scald/post burn contracture

Cardiomyopathy/cardiomegaly

Cellulitis

Cerebral atrophy/cortical atrophy

Cerebral malaria/malaria/severe malaria

Cerebral palsy

Chicken pox

Developmental delay

Diabetes/diabetes mellitus

Down's syndrome

Drug overdose/drug reaction/poisoning

Electrolyte imbalance

Empyema thoracis

Encephalopathy

Eventration of diaphragm

Extrapulmonary tuberculosis

Failure to thrive

Feeding problem/feeding mismanagement

Fistula

G-6-PD deficiency

Gastro esophageal reflux disease

Growing pain

Guillain-Barre syndrome/GBS

Gut atresia/choanal atresia/intestinal

Gut malrotation

Haemorrhagic diseases of newborn

HB-E diseases

Head injury

Healthy baby/normal baby/well baby

Heart failure

Hemangioma

Hematemesis

Hematoma/cephalohematoma

Hemiplegia

Hemolytic anemia

Hepatitis/neonatal hepatitis syndrome

Chocking/chocking attack
Cholecystitis/choledochal cyst
Chronic kidney disease/CKD
Chronic suppurative otitis media (CSOM)
CLD/chronic liver disease
Cleft lip/Cleft palate
Hydrocephalus
Hydronephrosis
Hypoglycemia
Hypospadias
Hypothyroidism
ICSOL/intracranial space occupying lesions
Ichthyosis
IDM/infant of diabetic mother
Infantile colic/infantile spasm
Infantile hypertrophic pyloric stenosis
Infected scabies/scabies
Infective endocarditis
Injury/cut injury/lacerated injury
Insect bite/bee bite
Intestinal obstruction/paralytic ileus
Intestinal perforation
Intestinal tuberculosis
Intrauterine growth retardation (IUGR)
Intussusception
ITP /idiopathic thrombocytopenic Purpura
Jaundice
Jejunal atresia
Kala azar

Hepatoblastoma
Hernia/inguinal hernia
Hypoxic-ischaemic encephalopathy
Hirschsprung disease/ HD
Hypertension
Hydrocele/ Encysted hydrocele
Per rectal bleeding
Pericardial effusion
Pharyngitis
Phimosis/para phimosis
Pierre robin syndrome
Pleural effusion
Pneumonic consolidation
Pneumonitis
Pneumothorax/Hydro pneumothorax
Polycystic kidney disease/ PKD
Portal hypertension
Post circumcision bleeding
Post measles pneumonia
Post meningitis neurological sequalae
Postdated baby
Pseudotumor cerebri
Pulmonary hypertension
Pure red cell aplasia
Posterior urethral valve
Pyrexia of unknown origin
Rabies
Reactive airway disease
Rectal polyp

Laryngomalacia
Lipoma
Liver abscess/amoebic liver abscess
Liver failure/hepatic failure
Low Birth weight (LBW)
Lung abscess
Lung collapse
Lymphadenitis/ lymphadenopathy
Lymphoma/ Hodgkin's disease
Measles
Meconium aspiration syndrome (MAS)
Medulloblastoma
Malingering
Meningocele/myelocele/ meningomyelitis
Meningoencephalitis
Microcephaly
Motor neuron disease/UMN
Mumps
Muscular atrophy/muscular dystrophy
Myopathy/ Myositis/myalgia
Near drowning
Neonatal convulsion/neonatal seizure
Nephroblastoma
Neuroblastoma
Neurodegenerative disorder
Neurometabolic disease
Omphalitis
Omphalocele
Oral thrush
Respiratory wheeze/recurrent wheeze
Rheumatic fever
Rickets
Rickettsial fever
Scarlet fever
Septic arthritis
Sequelae
Some/severe dehydration
Stevens Johnson Syndrome
Stomatitis/ Gingivostomatitis
Storage disease
Subdural effusion
Subacute sclerosing panencephalitis
Synovitis/transient synovitis
Teratoma
Transposition of great artery
Thalassemia
Tonsilitis
TORCH infection
Transient tachypnea of newborn
Tubercular meningitis
Tuberculosis/disseminated tuberculosis
Umbilical hernia
Umbilical sepsis
Undescendent testis
Upper respiratory tract infection
Urinary tract infection
Urine suppression
Urticaria/allergic reaction

Osteomyelitis/ acute Osteomyelitis
Pancreatitis/acute pancreatitis/chron
PEM/2° PEM
Peptic ulcer disease/PUD
Valvular heart disease
Whopping cough
Wilms tumor

Table S2. Reasons for sample collection failure

Reasons	Number of Cases	Percentage
Did not consent	152	14·6
Use of high flow oxygen canula,<br>Cpap/incubators/headbox/intubation	133	12·8
Moved to a special ward	64	6·1
Discharged before specimen collection	669	64·2
Other reasons	24	2·3

Table S3. Time of sample collection

Time of sample collection was available for 6119 of 6149 samples.

The interval between time of admission and sample collection	Number of samples collected (%)	Number of RSV-positive samples collected (%)
0 - 23 hours	2732 (44·6%)	537 (42·7%)
24 - 47 hours	2161 (35·3%)	467 (37·1%)
48 - 71 hours	717 (11·7%)	155 (12·3%)
>=72 hours	509 (8·3%)	99 (7·9%)

Table S4. Hazard ratio (HR) of denied vs admitted participants adjusted by age, sex, and diagnosis

*A list of diagnoses that may be included in "other" is listed in the table S1, appendix, page 19.

Adjusted by	Strata	HR [95% CI]
		1·69 [1·48, 1·94]
Age, Sex, Diagnosis		1·56 [1·34, 1·81]
Sex, Diagnosis	< 1 month	2·27 [1·87, 2·75]
Sex, Diagnosis	2-3 months	0·73 [0·53, 1·00]
Sex, Diagnosis	4 months - 1 year	1·27 [0·67, 2·40]
Sex, Diagnosis	1-5 years	0·71 [0·43, 1·16]
Age, Diagnosis	Male	1·76 [1·44, 2·15]
Age, Diagnosis	Female	1·30 [1·03, 1·63]
Age, Sex	Respiratory manifestation	0·95 [0·73, 1·23]
Age, Sex	Perinatal asphyxia	2·05 [1·59, 2·65]
Age, Sex	Systemic infections	0·82 [0·61, 1·09]
Age, Sex	Preterm low-birth-weight	2·78 [1·99, 3·87]
Age, Sex	Febrile illness	1·44 [0·20, 10·28]
Age, Sex	Gastrointestinal manifestation	2·16 [0·84, 5·54]
Age, Sex	Other*	1·35 [1·17, 1·56]

Table S5. Details of all recorded deceased RSV-positive cases

ID	Sex	Age (days)	Hospital duration (Days)	Hospital outcome	Follow up	Admission to death (Days)	Place of death	Final Diagnoses at study hospital
1	F	161	0	Died	Yes	0	Study hospital	Acute respiratory infection, Heart failure Severe malnutrition
2	M	121	1	Died	No	1	Study hospital	Cholecystitis, choledochal cyst, cholelithiasis, hepatic failure
3	F	227	2	Died	No	2	Study hospital	Pneumonia, Hypertension
4	M	145	3	Died	Yes	3	Study hospital	Pneumonia
5	M	152	3	Died	No	3	Study hospital	Septicaemia/sepsis, Pneumonia, Meningitis
6	F	62	4	Died	Yes	4	Study hospital	Pneumonia, Septicaemia, Failure to thrive
7	F	138	4	Died	No	4	Study hospital	Septicaemia/sepsis
8	M	89	4	Died	No	4	Study hospital	Pneumonia, Heart failure
9	F	243	4	Died	No	4	Study hospital	Pneumonia, Septicaemia/sepsis
10	F	72	5	Died	No	5	Study hospital	Pneumonia, congenital heart disease, Heart failure
11	M	18	6	Died	No	6	Study hospital	Congenital heart disease
12	F	104	6	Died	No	6	Study hospital	Strangulated hernia

13	F	91	8	Died	No	8	Study hospital	Pneumonia, Ventricular septal defect
14	F	123	8	LAMA	Yes	8	Road	Pneumonia
15	F	3	8	Died	Yes	8	Study hospital	Perinatal asphyxia, Neonatal jaundice, Neonatal sepsis
16	M	91	9	Died	Yes	9	Study hospital	Pneumonia, Septicaemia/sepsis, Failure to thrive
17	M	78	10	Died	Yes	10	Study hospital	Pneumonia
18	M	2	11	Died	No	11	Study hospital	Ventricular septal defect Cardiomyopathy, Cardiomegaly
19	M	234	13	Died	No	13	Study hospital	Seizure disorder
20	F	485	13	Died	No	13	Study hospital	Pneumonia, Developmental delay
21	M	138	14	Died	No	14	Study hospital	Pneumonia
22	F	140	14	Died	No	14	Study hospital	Pneumonia, Septicaemia/sepsis, Intestinal obstruction
23	F	218	14	Died	No	14	Study hospital	Pneumonia, Ventricular septal defect, Down's syndrome
24	F	3	25	Died	No	25	Study hospital	Perinatal asphyxia, Neonatal sepsis, Neonatal jaundice, congenital heart disease
25	M	590	26	Died	No	26	Study hospital	Pneumonia, Septicaemia/sepsis Developmental delay, Pleural effusion
26	M	228	4	LAMA	Yes	6	Home	Pneumonia

27	M	106	8	LAMA	Yes	18	Home	Pneumonia
28	M	109	11	Discharge	Yes	21	Home	Pneumonia, Laryngomalacia
29	M	17	7	Discharge	Yes	29	Home	Hypertension, Neonatal sepsis
30	F	254	7	Discharge	Yes	32	Hospital	Ventricular septal defect
31	M	39	6	LAMA	Yes	32	Home	Pneumonia, congenital heart disease
32	M	17	4	Discharge	Yes	43	Hospital	Pneumonia, Ventricular septal defect, Transposition of great artery
33	M	111	5	LAMA	Yes	50	Home	Pneumonia, congenital heart disease
34	F	12	7	Discharge	Yes	62	Hospital	Pneumonia, congenital heart disease
35	M	334	6	Discharge	Yes	70	Home	Pneumonia, Ventricular septal defect, Down's syndrome
36	F	499	10	Discharge	Yes	82	Home	Pneumonic consolidation, Pleural effusion

Supplementary References

1 Case definitions. https://www.who.int/teams/global-influenza-programme/global-respiratory-syncytial-virus-surveillance/case-definitions (accessed Jan 6, 2024).

2 Elalouf A, Wachtel G. Queueing Problems in Emergency Departments: A Review of Practical Approaches and Research Methodologies. *Oper Res Forum* 2021; **3**: 2.

3 Fitzgerald K, Pelletier L, Reznek MA. A Queue-Based Monte Carlo Analysis to Support Decision Making for Implementation of an Emergency Department Fast Track. *Journal of Healthcare Engineering* 2017; **2017**: 6536523.

4 McManus ML, Long MC, Cooper A, Litvak E. Queuing theory accurately models the need for critical care resources. *Anesthesiology* 2004; **100**: 1271–6.

5 Kampmann B, Madhi SA, Munjal I, *et al.* Bivalent Prefusion F Vaccine in Pregnancy to Prevent RSV Illness in Infants. *New England Journal of Medicine* 2023; **388**: 1451–64.

6 Ohuma EO, Moller A-B, Bradley E, *et al.* National, regional, and global estimates of preterm birth in 2020, with trends from 2010: a systematic analysis. *The Lancet* 2023; **402**: 1261–71.

7 Hammitt LL, Dagan R, Yuan Y, *et al.* Nirsevimab for Prevention of RSV in Healthy Late-Preterm and Term Infants. *New England Journal of Medicine* 2022; **386**: 837–46.
