# Supplementary material for: Health-care burden related to respiratory syncytial virus in a resource-constrained setting: a prospective observational study
Source: Lancet Glob Health. 2025 May 21;13(6):e1072–81. doi: 10.1016/S2214-109X(25)00048-8 (PMC12100462; doi:10.1016/S2214-109X(25)00048-8)
Supplement: Equitable Partnership Declaration [file mmc2.pdf]

THE LANCET
Global Health

Supplementary appendix 2

This Equitable Partnership Declaration (EPD) was submitted by the authors, and we reproduce it as supplied. It has not been peer reviewed. *The Lancet*'s editorial processes have not been applied to the EPD.

Supplement to: Saha Se, Saha Su, Kanon N, et al. Health-care burden related to respiratory syncytial virus in a resource-constrained setting: a prospective observational study. *Lancet Glob Health* 2025; **13:** e1072–81.

Equitable Partnership Declaration questions

This Equitable Partnership Declaration is a statement being published online alongside papers at *The Lancet Global Health*, as a separate appendix, to allow researchers to describe how their work engages with researchers, communities, and environments in the countries of study. This is part of our broader goal to decolonise global health, handing control and leadership of research to academics and clinicians who are based in the regions of study, and to affected communities.

Please answer all questions with as much detail as possible, noting that all included information will be published open-access and it will be freely available online to all who wish to read it. If a question does not apply to your study, please state "Not applicable".

The format of and questions in this statement are currently in a pilot phase. Please email Dr Liam Messin (Liam.Messin@lancet.com; deputy editor) and Dr Kate McIntosh (Kate.McIntosh@lancet.com; senior editor) with any feedback, particularly if you find any questions unclear.

Researcher considerations

1. Please detail the involvement that researchers who are based in the region(s) of study had during a) study design; b) clinical study processes, such as processing blood samples, prescribing medication, or patient recruitment; c) data interpretation; and d) manuscript preparation, commenting on all aspects. If they were not involved in any of these aspects, please explain why.

   This question is intended for international partnerships; if all your authors are based in the area of study, this question is not applicable.

   This should include a thorough description of their leadership role(s) in the study. Are local researchers named in the author list or the acknowledgements, or are they not mentioned at all (and, if not, why)? Please also describe the involvement of early career researchers based in the location of the study. Some of this information might be repeated from the Contributors section in the manuscript. Note: we adhere to ICMJE authorship criteria when deciding who should be named on a paper.

**NA**
**NA**
**NA**
**NA**

2. Were the data used in your study collected by authors named on the paper, or have they been extracted from a source such as a national survey? ie, is this a secondary analysis of data that were

not collected by the authors of this paper. If the authors of this paper were not involved in data collection, how were data interpreted with sufficient contextual knowledge?

The Lancet Global Health *believe contextual understanding is crucial for informed data analysis and interpretation.*

The data used in this study were prospectively collected by the authors as part of an observational surveillance study. No external data sources were used. The researchers' (who are based in the study hospital and the local research foundation) deep understanding of the local context ensured accurate and meaningful interpretation of findings.

3. How was funding used to remunerate and enhance the skills of researchers and institutions based in the area(s) of study? And how was funding used to improve research infrastructure in the area of study?

   Potentially effective investments into long-term skills and opportunities within institutions could include training or mentorship in analytical techniques and manuscript writing, opportunities to lead all or specific aspects of the study, financial remuneration rather than requiring volunteers, and other professional development and educational opportunities.

   Improvements to research infrastructure could be funding of extended trial designs (such as platform trials) and use of master protocols to enable these designs, establishment of long-term contracts for research staff, building research facilities, and local control of funding allocation.

Funding was utilized to provide salaries and comprehensive training in sample collection, qPCR testing, data analysis, and manuscript writing. Mentorship opportunities were provided for early-career researchers (NK, YH, MSI) enabling them to lead aspects of the study. Most substantial skills developed through this project were the data analysis skills.

The funding supported research infrastructure development, including a laboratory equipment upgrades (e.g., refrigerator) and data management systems.

4. How did you safeguard the researchers who implemented the study?

Please describe how you guaranteed safe working conditions for study staff, including provision of appropriate personal protective equipment, protection from violence, and prevention of overworking.

Laboratory researchers and staff were provided with all the required training and personal protective equipment, ensuring safe working conditions with clinical samples. Work schedules were managed to prevent overworking, and the study adhered to rigorous safety protocols.

<u>Benefits to the communities and regions of study</u>

5. How does the study address the research and policy priorities of its location?

How were the local priorities determined and then used to inform the research question? Who decided which priorities to take forward? Which elements of the study address those priorities?

The study addressed critical gaps in understanding RSV burden in Bangladesh, a country with a high infectious disease burden and limited healthcare resources. Priorities were identified through consultations with healthcare providers, and CHRF's long standing involvement in local healthcare research.

6. How will research products be shared in the community of study?

For instance, will you be providing written or oral layperson summaries for non-academic information sharing? Will study data be made available to institutions in the region(s) of study? The Lancet Global Health *encourages authors to translate the summary (abstract) into relevant languages after paper editing; do you intend to translate your summary?*

The findings will be disseminated through academic publications, blog/social media posts and translated summaries in Bangla to ensure accessibility to policymakers and the public. Layperson summaries will be shared with public health stakeholders.

7. How were individuals, communities, and environments protected from harm?

a) *How did you ensure that sensitive patient data was handled safely and respectfully? Was there any potential for stigma or discrimination against participants arising from any of the procedures or outcomes of the study?*

Sensitive patient data were securely handled to maintain confidentiality. All data were de-identified by trained personnel and data managers before analysis and sharing with other authors. There was no potential for stigma or discrimination against participants arising from any of the procedures or outcomes of the study.

b) *Might any of the tests be experienced as invasive or culturally insensitive?*

To the best of our knowledge, none of the tests were experienced as invasive or culturally insensitive.

c) *How did you determine that work was sensitive to traditions, restrictions, and considerations of all cultural and religious groups in the study population?*

The protocol of the study was approved by an ethical review board that includes members from diverse background (e.g. religious leader) to ensure considerations of all cultural and religious groups in the study population.

e) *Were biowaste and radioactive waste disposed of in accordance with local laws?*

All biowaste was disposed in accordance with local laws.

f) *Were any structures built that would have impacted members of the community or the environment (such as handwashing facilities in a public space)? If so, how did you ensure that you had appropriate community buy-in?*

NA

g) *How might the study have impacted existing health-care resources (such as staff workloads, use of equipment that is typically employed elsewhere, or reallocation of public funds)?*

The study minimized its impact on healthcare resources by hiring dedicated research staff funded through the project, ensuring no additional burden on hospital personnel. Laboratory equipment for the study was independently procured, avoiding the use of hospital diagnostics. No public

funds or hospital budgets were reallocated. The study aimed to address resource strain rather than exacerbate it, providing data to advocate for improved healthcare capacity in Bangladesh.

8. Finally, please provide the title (eg, Dr/Prof, Mr/Mrs/Ms/Mx), name, and email address of an author who can be contacted about this statement. This can be the corresponding author.

Name: Dr. Senjuti Saha
Email: senjutisaha@chrfbd.org
